# Dataset on microstructural characteristics and mechanical performance of homogeneous and functionally graded fibrous scaffolds

**DOI:** 10.1016/j.dib.2019.104718

**Published:** 2019-10-28

**Authors:** Weily Khoo, She Man Chung, Shing Chee Lim, Cheng Yee Low, Jenna M. Shapiro, Ching Theng Koh

**Affiliations:** a Faculty of Mechanical and Manufacturing Engineering, University of Tun Hussein Onn Malaysia, Johor, Malaysia; b Faculty of Engineering, University of Bristol, Bristol, United Kingdom

**Keywords:** Electrospinning, Gelatin scaffold, Thickness, Tensile properties, Fracture properties, Finite element analysis, Functionally graded materials, Fibrous materials

## Abstract

Data in this article are supplementary to the corresponding research article [1]. Morphological features of homogeneous and graded nanofibrous electrospun gelatin scaffolds were observed using scanning electron microscopy. Microstructural properties including fiber diameter and pore size were determined via image analysis, using ImageJ. Uniaxial tensile and fracture tests were performed on both homogeneous and graded scaffolds using a universal testing machine. Stress-strain curves of all scaffolds are presented. Computing software, MATLAB, was used to design fibrous networks with thickness-dependent density and alignment gradients (DAG). Finite element analysis software, Abaqus, was used to determine the effect of the number of layers on the fracture properties of DAG multilayer scaffolds.

**Table:** Specifications Table

Subject	Materials Science, Engineering
Specific subject area	Electrospinning, Tensile properties, Fracture properties, Fibrous materials
Type of data	Table and figure
How data were acquired	• Scanning electron microscope (SEM, Hitachi) • Digital calipers (Facom, France) • Universal testing machine (Lloyd Instruments Ltd, UK) • ImageJ (NIH, Bethesda, MD, USA) • MATLAB (Version 2017, MathWorks, Natick, MA, USA) • Abaqus (Version 2017, SIMULIA, Providence, RI, USA)
Data format	Raw and analyzed
Parameters for data collection	The morphology of individual layers of graded scaffolds was visualized after each layer was completely spun.
Description of data collection	For fiber diameter and pore size determination, measurements were taken via ImageJ of ten individual fibers and pores in each SEM micrograph, and the average measurements were used. For thickness determination, four to six test samples were used. Three different points of each test sample were measured. For uniaxial and fracture tests, four to six test samples were used for each test.
Data source location	University Tun Hussein Onn Malaysia, Parit Raja, Malaysia.
Data accessibility	Data are available with this article
Related research article	Weily Khoo, She Man Chung, Shing Chee Lim, Cheng Yee Low, Jenna M. Shapiro and Ching Theng Koh, “Fracture Behavior of Multilayer Fibrous Scaffolds Featuring Microstructural Gradients”, Materials & Design, Under Review [1].

**Table:** 

**Value of the Data** • Multilayer fibrous structures with microstructural gradients have many potential uses in a variety of applications, including filtration and tissue engineering. • Microstructural characteristics and mechanical performance of homogeneous and graded scaffolds are presented. These data could benefit researchers in material science, mechanical engineering and tissue engineering. • The data presented give insights in determining an appropriate number of layers for desired fracture behavior when designing multilayer fibrous scaffolds with microstructural gradients in fiber density and fiber alignment.

## 1 Data

**Fig. 1:**
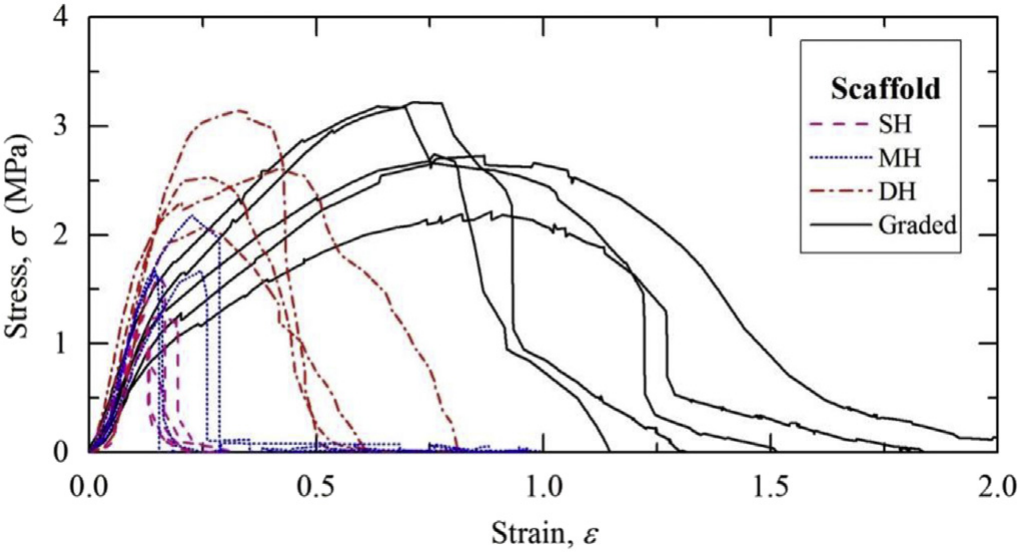
Stress-strain curves of homogeneous and graded electrospun scaffolds obtained via uniaxial tensile test.

**Fig. 2:**
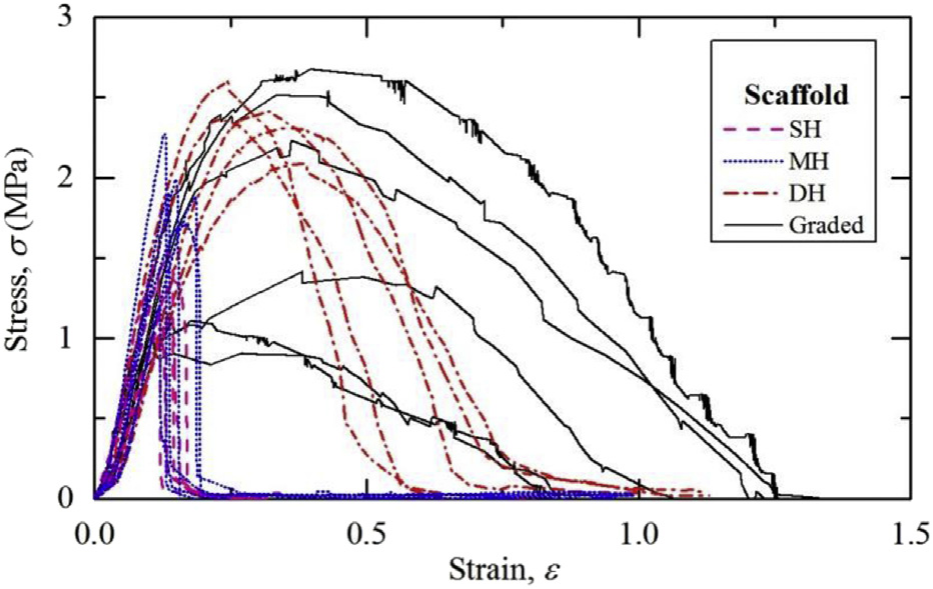
Stress-strain curves of homogeneous and graded electrospun scaffolds obtained via fracture test.

**Fig. 3:**
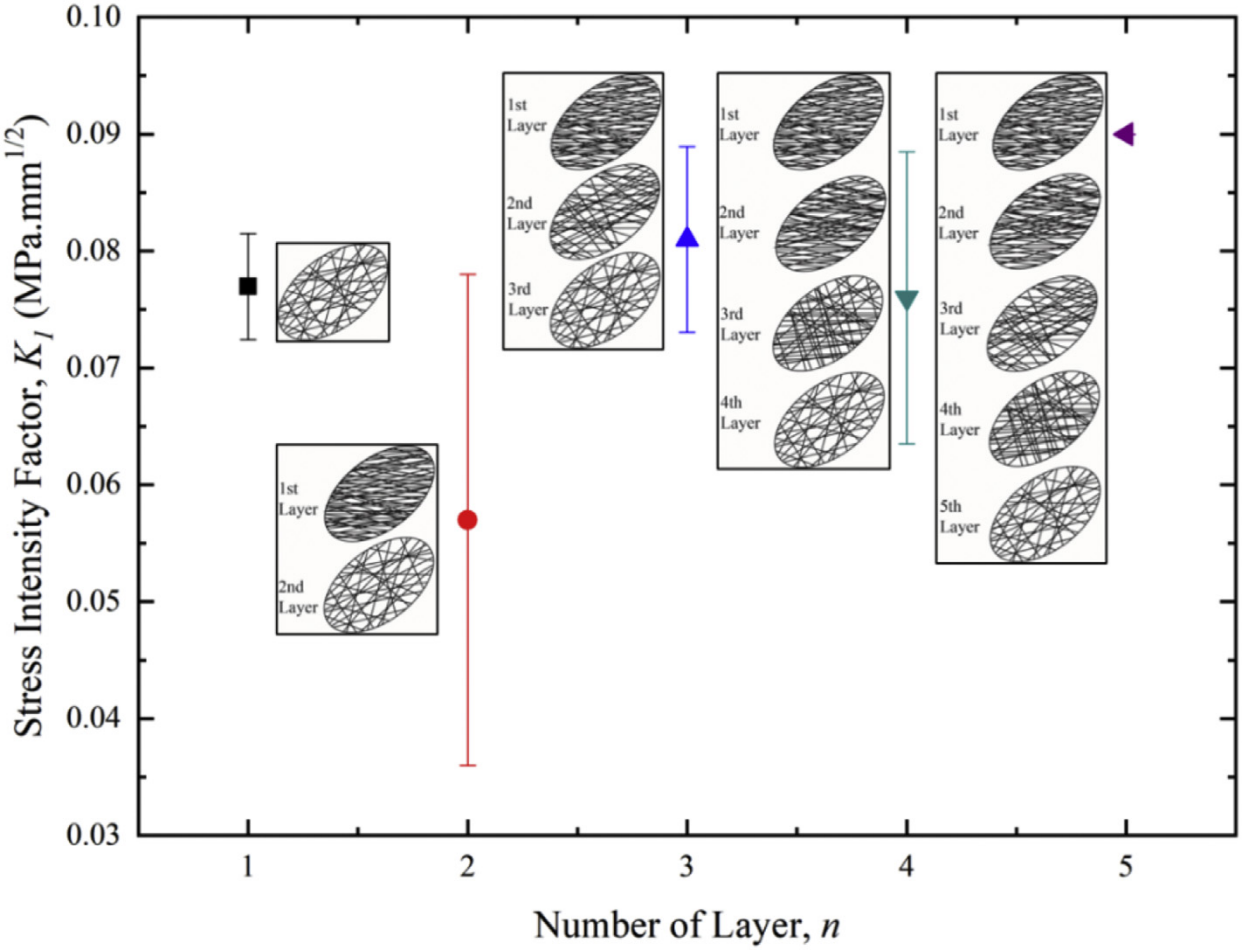
Stress intensity factor for DAG networks with varying numbers of layers corresponding to different levels of network density gradients and fiber alignment gradients.

The microstructural properties of homogeneous and graded electrospun scaffolds are tabulated in Table 1 (fiber diameter) and Table 2 (pore size). Thickness measurements of homogeneous and graded electrospun scaffolds are listed in Table 3. The stress-strain curves of all electrospun scaffolds are plotted in Fig. 1 (uniaxial tensile test) and Fig. 2 (fracture test). In order to study the effect of layering on fracture properties of graded fibrous networks, computational analyses were performed. Fibrous networks featuring density and alignment gradients were modeled with up to five layers (Table 4). The effect of number of layers on stress intensity factor is presented in Fig. 3.

**Table 1:** Fiber diameter measurement on homogeneous and graded electrospun scaffolds.

	Homogeneous scaffolds			Graded scaffolds		
	DH	MH	SH	Bottom layer	Middle layer	Top layer
**Fiber diameter (nm)**	208	504	779	255	316	717
	197	357	863	238	387	662
	228	423	711	226	287	667
	217	562	663	228	408	730
	174	450	578	202	436	667
	219	369	782	244	496	666
	200	417	909	233	371	663
	191	493	806	188	398	629
	210	442	586	204	385	765
	208	344	713	253	454	620
**Average**	205	436	739	227	394	679
**Standard deviation**	15	70	110	23	62	45

**Table 2:** Pore size measurement of homogeneous and graded electrospun scaffolds.

	Homogeneous scaffolds			Graded scaffolds		
	DH	MH	SH	Bottom layer	Middle layer	Top layer
**Pore size (μm**^**2**^**)**	0.755	2.604	5.084	1.260	3.536	4.243
	1.342	1.728	10.190	1.193	3.103	3.447
	0.715	1.925	7.390	1.407	2.271	3.309
	0.557	2.570	6.485	0.924	3.931	2.884
	0.395	3.322	4.960	1.059	1.322	8.347
	0.641	3.615	3.872	0.645	2.468	2.864
	0.750	6.434	6.135	1.550	1.046	3.508
	0.757	2.373	4.999	1.170	1.710	7.037
	1.056	2.606	15.858	1.326	2.066	6.462
	0.464	1.875	9.027	0.837	4.526	7.193
**Average**	0.743	2.905	7.400	1.137	2.598	4.929
**Standard deviation**	0.279	1.379	3.558	0.275	1.148	2.091

**Table 3:** Thickness of homogeneous and graded electrospun scaffolds.

Type of scaffolds	Thickness (mm)			
	Reading 1	Reading 2	Reading 3	Average
**SH scaffolds**	0.17	0.29	0.34	0.27
	0.34	0.33	0.30	0.32
	0.17	0.28	0.30	0.25
	0.29	0.30	0.29	0.29
	0.17	0.25	0.31	0.24
	0.36	0.32	0.32	0.33
	0.10	0.09	0.10	0.10
	0.08	0.07	0.08	0.08
	0.08	0.08	0.09	0.08
	0.07	0.07	0.06	0.07
	0.08	0.09	0.09	0.09
**MH scaffolds**	0.08	0.11	0.10	0.10
	0.19	0.20	0.19	0.19
	0.22	0.24	0.22	0.23
	0.2	0.22	0.25	0.22
	0.12	0.16	0.17	0.15
	0.13	0.16	0.18	0.16
	0.07	0.07	0.07	0.07
	0.14	0.16	0.15	0.15
	0.14	0.16	0.19	0.16
	0.10	0.09	0.04	0.08
	0.07	0.09	0.12	0.09
**DH scaffolds**	0.16	0.19	0.20	0.18
	0.21	0.21	0.19	0.20
	0.17	0.18	0.18	0.18
	0.18	0.19	0.20	0.19
	0.18	0.21	0.23	0.21
	0.13	0.14	0.15	0.14
	0.10	0.11	0.12	0.11
	0.10	0.09	0.09	0.09
	0.15	0.13	0.09	0.12
**Graded scaffolds**	0.61	0.61	0.62	0.61
	0.70	0.67	0.70	0.69
	0.54	0.55	0.61	0.57
	0.34	0.30	0.30	0.31
	0.41	0.42	0.44	0.42
	0.41	0.43	0.44	0.43
	0.70	0.69	0.68	0.69
	0.69	0.72	0.70	0.70
	0.62	0.66	0.62	0.63
	0.48	0.50	0.55	0.51
	0.40	0.44	0.44	0.43

**Table 4:** Network parameters of DAG networks constructed with various number of layers.

Number of Layer, *n*	Fiber Density, *ρ*_*f (1st layer)*_ (μm^−1^)	Density Gradient, G (%)	Fiber Alignment, *Ø* (°)	Model Size, *r* (*μ*m)
1	36.3 ± 0.5	0	90	5
2	67.5 ± 1.3	52 ± 2	15, 90	5
3	68.3 ± 1.6	29 ± 2	15, 52.5, 90	5
4	68.1 ± 1.5	19 ± 3	15, 52.5, 65, 90	5
5	68.7 ± 0.7	16 ± 2	15, 33.75, 52.5, 71.25, 90	5

For further interpretation and discussion on the experimental dataset, readers are encouraged to refer the research article [1].

## 2 Experimental design, materials, and methods

### 2.1 Preparation of homogeneous and graded electrospun scaffolds

Fish skin gelatin (Sigma Aldrich, USA) and glacial acetic acid (Merck, Germany) were used in preparing a 25 wt % gelatin solution in a mixture of 90 wt % glacial acetic acid and 10 wt % water. Homogeneous and graded scaffolds were produced with a bespoke electrospinning set-up. Detailed preparation of electrospun scaffolds has been reported in the corresponding research article [1].

### 2.2 Morphology observation and quantification

Morphology of electrospun scaffolds were visualized using scanning electron microscope (SEM, Hitachi) at an accelerating voltage of 10 kV. Prior to SEM observation, each scaffold was cut into 10 mm × 10 mm squares and gold coated. SEM images were captured at magnification ×6000.

ImageJ (NIH, Bethesda, MD, USA) was used to measure fiber diameter and pore size of scaffolds. The average fiber diameters and pore sizes were determined by measuring and averaging the diameters of ten randomly chosen fibers and pore sizes respectively from one SEM image for each type of scaffolds. Individual fiber diameter and pore size measurements of homogeneous and graded electrospun scaffolds are shown in Table 1, Table 2, respectively.

### 2.3 Mechanical testing

Uniaxial tensile and fracture tests were performed on homogeneous and graded scaffolds. All mechanical test samples were cut to rectangles 24 mm in width and 3 mm in height. For fracture test samples, an 8 mm notch was introduced perpendicular to loading direction. Thickness of each test sample was measured at three separate points (center and both ends) using digital calipers (Facom, France). All measurements were averaged to determine mean thickness. The thickness data are tabulated in Table 3.

All the samples were gripped along their width and extended at a rate of 3mm/min until failure. Stress-strain responses of homogeneous and graded scaffolds are presented in Fig. 1 (uniaxial tensile) and Fig. 2 (fracture test). For each mechanical test, four to six test samples were used.

### 2.4 Computational analysis

#### 2.4.1 Finite element modeling

Fibrous networks composed of density and alignment gradients (DAG) were generated using MATLAB (Version 2017, MathWorks, Natick, MA, USA). Table 4 listed the parameters used to generate DAG networks consisting of up to five layers. The networks were then imported into finite element software, Abaqus (Version 2017, SIMULIA, Providence, RI, USA) for modeling. Fibers were modeled with Timoshenko beam (B31) and were analyzed using nonlinear finite element analysis, which considers large strain and rotation. All fibers were defined with diameter *Ø* of 300 nm, Young's modulus, *E*, of 100 MPa and fracture strength, *σ*_*f*_, of 30 ± 4 MPa.
